# The genome sequence of the thickback sole, *Microchirus variegatus *(Donovan, 1808)

**DOI:** 10.12688/wellcomeopenres.21139.1

**Published:** 2024-03-19

**Authors:** Rachel Brittain, Patrick Adkins, Robert J. Mrowicki, Joanna Harley, Vengamanaidu Modepali, Marine Biological Association Genome Acquisition Lab, Darwin Tree of Life Barcoding collective, Wellcome Sanger Institute Tree of Life Management, Samples and Laboratory team, Wellcome Sanger Institute Scientific Operations: Sequencing Operations, Wellcome Sanger Institute Tree of Life Core Informatics team, Tree of Life Core Informatics collective, Darwin Tree of Life Consortium

**Affiliations:** 1 The Marine Biological Association, Plymouth, England, UK

**Keywords:** Microchirus variegatus, thickback sole, genome sequence, chromosomal, Pleuronectiformes

## Abstract

We present a genome assembly from an individual female *Microchirus variegatus* (the thickback sole; Chordata; Actinopteri; Pleuronectiformes; Soleidae). The genome sequence is 724.7 megabases in span. Most of the assembly is scaffolded into 23 chromosomal pseudomolecules. The mitochondrial genome has also been assembled and is 17.42 kilobases in length.

## Species taxonomy

Eukaryota; Opisthokonta; Metazoa; Eumetazoa; Bilateria; Deuterostomia; Chordata; Craniata; Vertebrata; Gnathostomata; Teleostomi; Euteleostomi; Actinopterygii; Actinopteri; Neopterygii; Teleostei; Osteoglossocephalai; Clupeocephala; Euteleosteomorpha; Neoteleostei; Eurypterygia; Ctenosquamata; Acanthomorphata; Euacanthomorphacea; Percomorphaceae; Carangaria; Pleuronectiformes; Pleuronectoidei; Soleidae; *Microchirus*; *Microchirus variegatus* Donovan, 1808 (NCBI:txid90074).

## Background

*Microchirus variegatus* ( Donovan *et al.*, 1802), also known as the thickback sole or lucky sole, is a flatfish belonging to the Soleidae family. It is distributed in the north-east Atlantic Ocean and the Mediterranean Sea ( Amara *et al.*, 1998; Tous *et al.*, 2015). The thickback sole has a slender and elongated oval body shape, but is thicker from side to side. The thickback sole is among the smaller-sized sole species, typically measuring 12 to 15 cm, but can reach up to 20 cm for the older age classes of 10 to 13 years old ( Déniel, 1981). Spawning occurs in springtime, and unlike common soles, the juveniles of thickback soles settle offshore and then migrate inshore ( Amara *et al.*, 1998). The thickback sole species is benthic and can be located on sea-beds consisting of sand and mud, with a preference for coarse sand. It feeds on a wide range of small benthic organisms, mainly crustaceans such as amphipods and shrimp, as well as polychaetes and bivalves.

Less commercially valuable than other flatfish species ( Machado *et al.*, 2020), *M. varigatus* is currently listed as of least concern by the IUCN red list and is a somewhat common species in its given distributions ( Tous *et al.*, 2015). This chromosome-level reference genome for *Microchirus variegatus* is the first for this species, and will provide the tools for further study into this species.

## Genome sequence report

**Figure 1.**
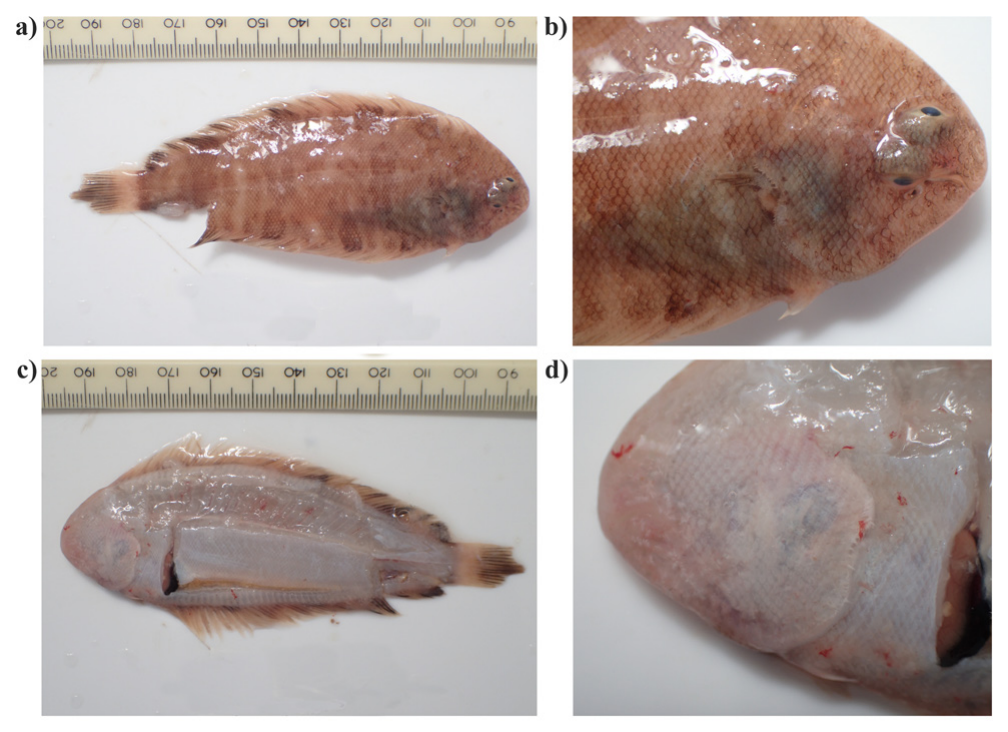
Photographs of the *Microchirus variegatus* (fMicVar1) specimen used for genome sequencing: **a**) View of the right side of the whole animal, **b**) View of the head of the right side of the animal, **c**) View of the left side of the whole animal, **d**) View of the head of the left side of the animal.

The genome was sequenced from one female *Microchirus variegatus* ( Figure 1) collected from RV MBA Sepia (50.25, –4.22). A total of 36-fold coverage in Pacific Biosciences single-molecule HiFi long reads was generated. Primary assembly contigs were scaffolded with chromosome conformation Hi-C data. Manual assembly curation corrected 22 missing joins or mis-joins, reducing the scaffold number by 3.59%.

**Figure 2.**
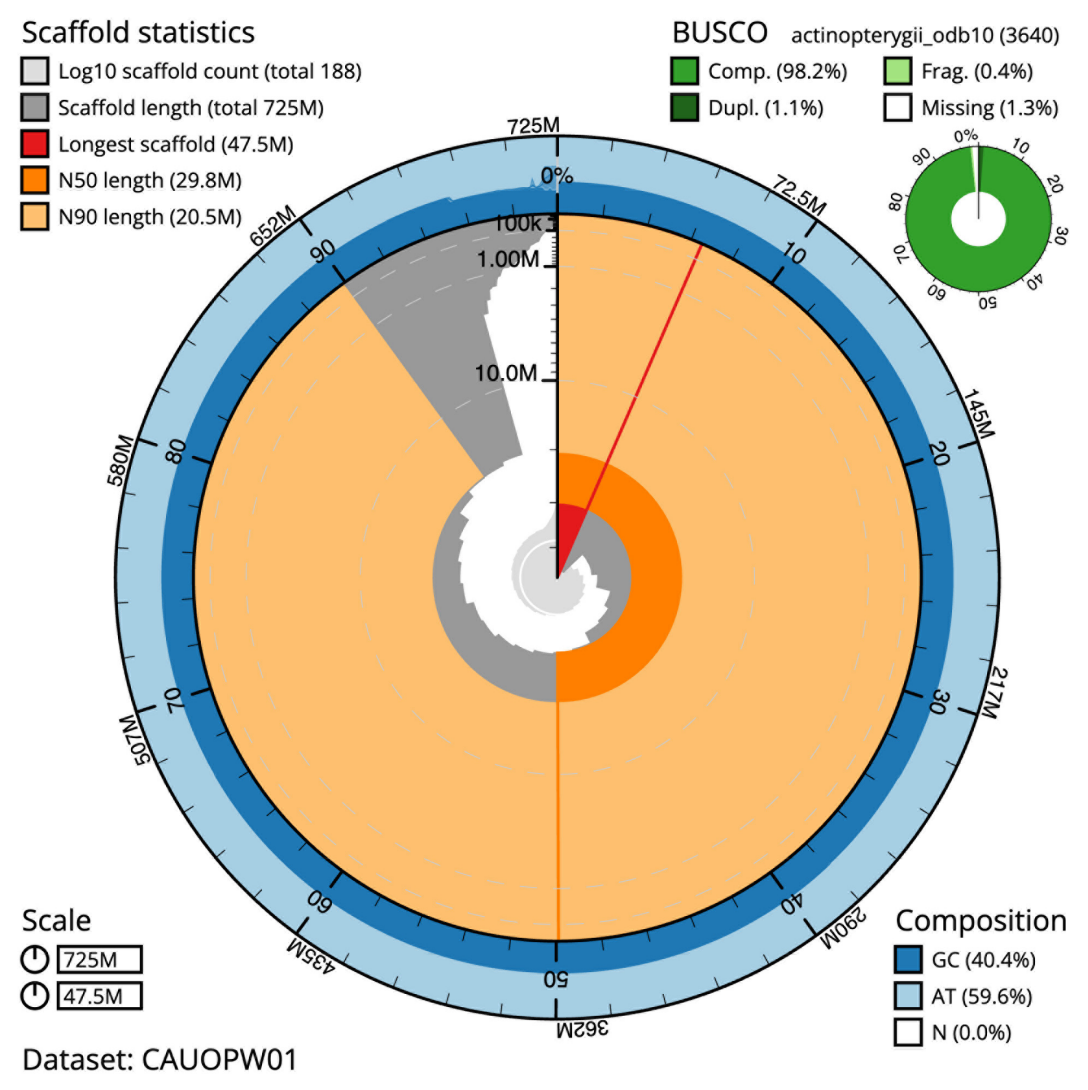
Genome assembly of *Microchirus variegatus*, fMicVar1.1: metrics. The BlobToolKit snail plot shows N50 metrics and BUSCO gene completeness. The main plot is divided into 1,000 size-ordered bins around the circumference with each bin representing 0.1% of the 724,694,306 bp assembly. The distribution of scaffold lengths is shown in dark grey with the plot radius scaled to the longest scaffold present in the assembly (47,546,321 bp, shown in red). Orange and pale-orange arcs show the N50 and N90 scaffold lengths (29,835,075 and 20,522,917 bp), respectively. The pale grey spiral shows the cumulative scaffold count on a log scale with white scale lines showing successive orders of magnitude. The blue and pale-blue area around the outside of the plot shows the distribution of GC, AT and N percentages in the same bins as the inner plot. A summary of complete, fragmented, duplicated and missing BUSCO genes in the actinopterygii_odb10 set is shown in the top right. An interactive version of this figure is available at https://blobtoolkit.genomehubs.org/view/CAUOPW01/dataset/CAUOPW01/snail.

**Figure 3.**
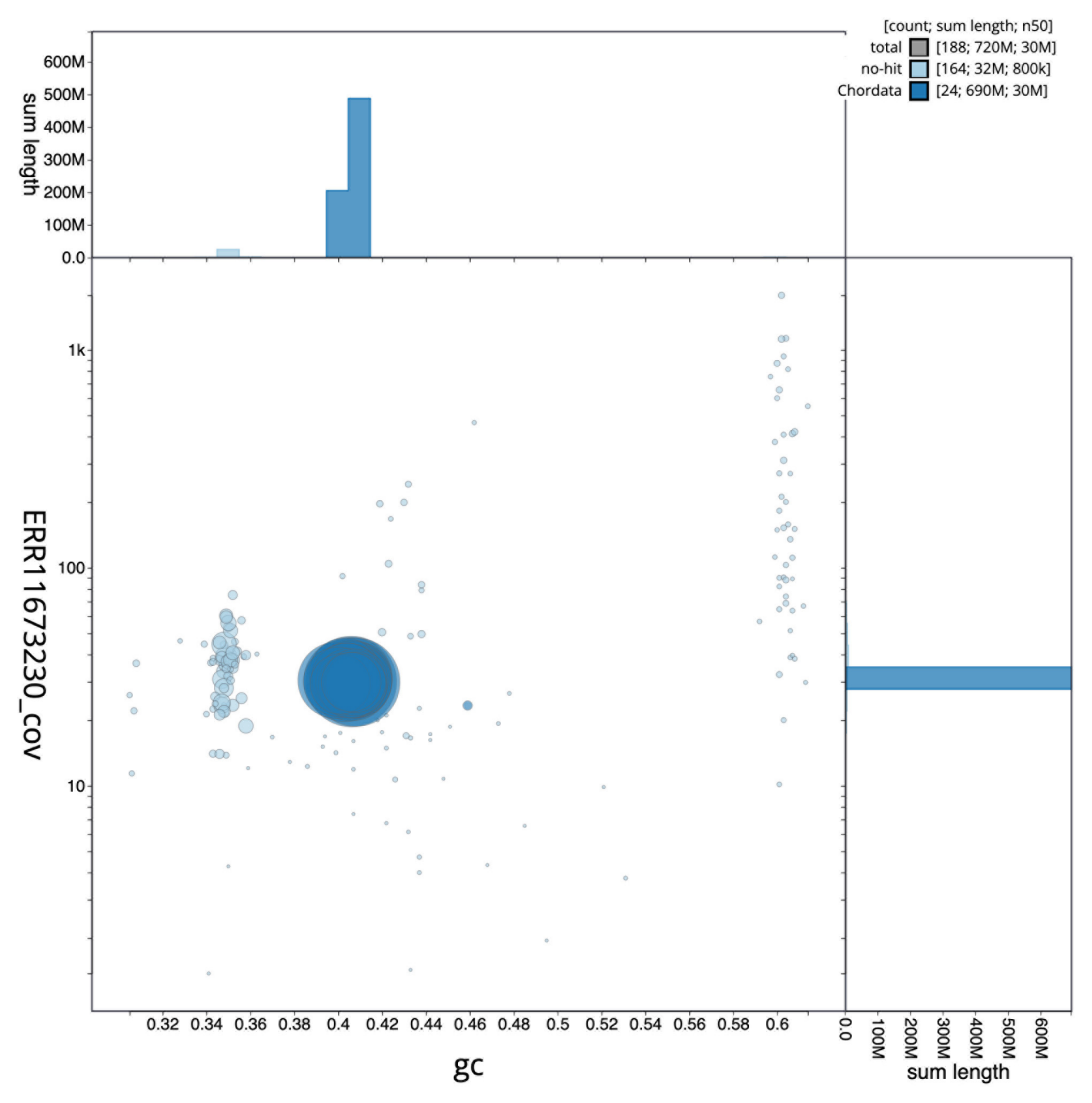
Genome assembly of *Microchirus variegatus*, fMicVar1.1: BlobToolKit GC-coverage plot. Sequences are coloured by phylum. Circles are sized in proportion to sequence length. Histograms show the distribution of sequence length sum along each axis. An interactive version of this figure is available at https://blobtoolkit.genomehubs.org/view/CAUOPW01/dataset/CAUOPW01/blob.

**Figure 4.**
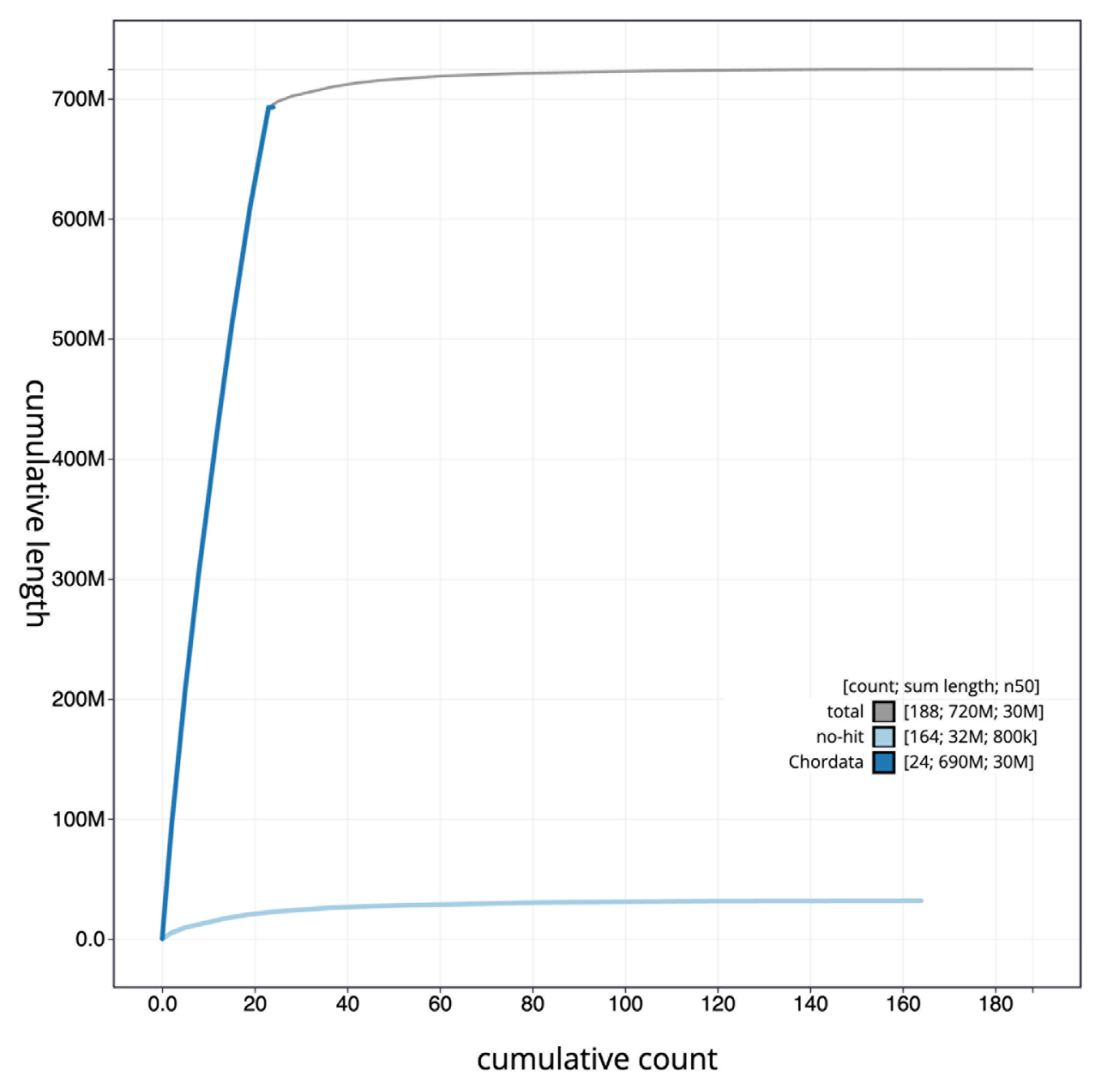
Genome assembly of *Microchirus variegatus*, fMicVar1.1: BlobToolKit cumulative sequence plot. The grey line shows cumulative length for all sequences. Coloured lines show cumulative lengths of sequences assigned to each phylum using the buscogenes taxrule. An interactive version of this figure is available at https://blobtoolkit.genomehubs.org/view/CAUOPW01/dataset/CAUOPW01/cumulative.

**Figure 5.**
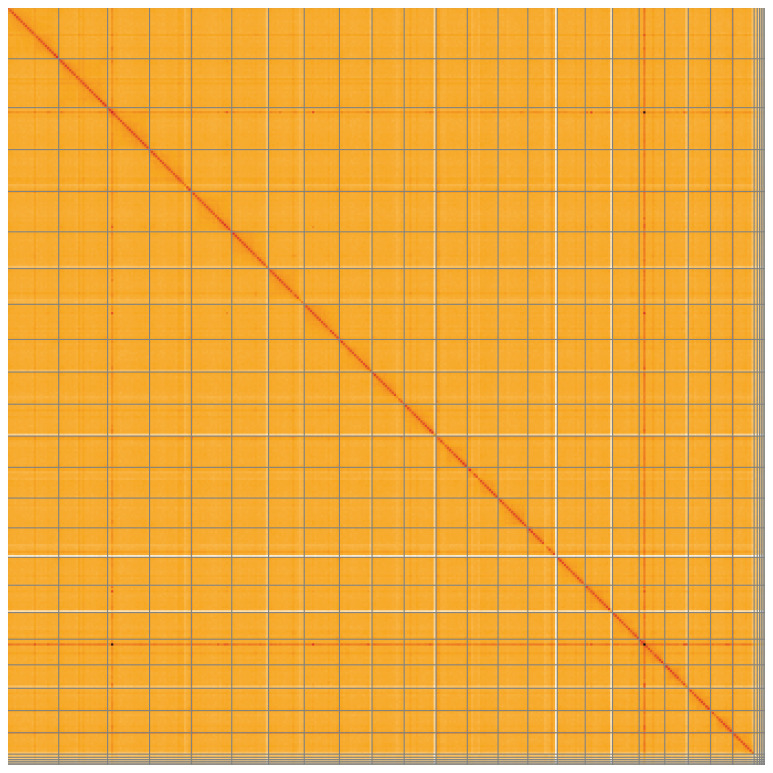
Genome assembly of *Microchirus variegatus*, fMicVar1.1: Hi-C contact map of the fMicVar1.1 assembly, visualised using HiGlass. Chromosomes are shown in order of size from left to right and top to bottom. An interactive version of this figure may be viewed at https://genome-note-higlass.tol.sanger.ac.uk/l/?d=NshZEA2dQ8e4VXrq_Qlgow.

The final assembly has a total length of 724.7 Mb in 187 sequence scaffolds with a scaffold N50 of 29.8 Mb ( Table 1). The snail plot in Figure 2 provides a summary of the assembly statistics, while the distribution of assembly scaffolds on GC proportion and coverage is shown in Figure 3. The cumulative assembly plot in Figure 4 shows curves for subsets of scaffolds assigned to different phyla. Most (95.61%) of the assembly sequence was assigned to 23 chromosomal-level scaffolds. Chromosome-scale scaffolds confirmed by the Hi-C data are named in order of size ( Figure 5; Table 2). While not fully phased, the assembly deposited is of one haplotype. Contigs corresponding to the second haplotype have also been deposited. The mitochondrial genome was also assembled and can be found as a contig within the multifasta file of the genome submission.

**Table 1.** Genome data for *Microchirus variegatus*, fMicVar1.1.

Project accession data		
Assembly identifier	fMicVar1.1	
Species	*Microchirus variegatus*	
Specimen	fMicVar1	
NCBI taxonomy ID	90074	
BioProject	PRJEB64068	
BioSample ID	SAMEA12219470	
Isolate information	fMicVar1, female: muscle (DNA and RNA sequencing), muscle and gill tissue (Hi-C sequencing)	
Assembly metrics *		*Benchmark*
Consensus quality (QV)	59.4	*≥ 50*
*k*-mer completeness	100.0%	*≥ 95%*
BUSCO **	C:98.2%[S:97.1%,D:1.1%], F:0.4%,M:1.3%,n:3,640	*C ≥ 95%*
Percentage of assembly mapped to chromosomes	95.61%	*≥ 95%*
Sex chromosomes	None	*localised homologous pairs*
Organelles	Mitochondrial genome: 17.42 kb	*complete single alleles*
Raw data accessions		
PacificBiosciences SEQUEL II	ERR11673230	
Hi-C Illumina	ERR11679381, ERR11679383	
PolyA RNA-Seq Illumina	ERR11679382	
Genome assembly		
Assembly accession	GCA_963457635.1	
*Accession of alternate haplotype*	GCA_963457655.1	
Span (Mb)	724.7	
Number of contigs	642	
Contig N50 length (Mb)	2.4	
Number of scaffolds	187	
Scaffold N50 length (Mb)	29.8	
Longest scaffold (Mb)	47.55	

* Assembly metric benchmarks are adapted from column VGP-2020 of “Table 1: Proposed standards and metrics for defining genome assembly quality” from Rhie *et al.* (2021). ** BUSCO scores based on the actinopterygii_odb10 BUSCO set using version 5.3.2. C = complete [S = single copy, D = duplicated], F = fragmented, M = missing, n = number of orthologues in comparison. A full set of BUSCO scores is available at https://blobtoolkit.genomehubs.org/view/CAUOPW01/dataset/CAUOPW01/busco.

**Table 2.** Chromosomal pseudomolecules in the genome assembly of *Microchirus variegatus*, fMicVar1.

INSDC accession	Chromosome	Length (Mb)	GC%
OY735174.1	1	47.55	40.5
OY735175.1	2	45.35	41.0
OY735176.1	3	38.9	40.5
OY735177.1	4	38.82	40.5
OY735178.1	5	37.45	40.5
OY735179.1	6	34.15	40.5
OY735180.1	7	33.11	40.0
OY735181.1	8	32.62	40.5
OY735182.1	9	30.22	40.5
OY735183.1	10	29.84	41.0
OY735184.1	11	29.57	40.5
OY735185.1	12	29.01	40.5
OY735186.1	13	28.49	40.5
OY735187.1	14	27.62	40.5
OY735188.1	15	27.32	40.0
OY735189.1	16	25.89	40.5
OY735190.1	17	25.38	40.5
OY735191.1	18	24.7	40.5
OY735192.1	19	23.78	41.0
OY735193.1	20	21.78	41.0
OY735194.1	21	20.65	41.0
OY735195.1	22	20.52	40.0
OY735196.1	23	19.89	40.5
OY735197.1	MT	0.02	47.0

The estimated Quality Value (QV) of the final assembly is 59.4 with *k*-mer completeness of 100.0%, and the assembly has a BUSCO v5.3.2 completeness of 98.2% (single = 97.1%, duplicated = 1.1%), using the actinopterygii_odb10 reference set ( *n* = 3,640).

Metadata for specimens, barcode results, spectra estimates, sequencing runs, contaminants and pre-curation assembly statistics are given at https://tolqc.cog.sanger.ac.uk/darwin/fish/Microchirus_variegatus/.

## Methods

### Sample acquisition and nucleic acid extraction

A female *Microchirus variegatus* (specimen ID MBA-210611-004A, ToLID fMicVar1) was taken using an Agassiz trawl deployed from RV MBA Sepia (latitude 50.25, longitude –4.22) on 2021-06-11. The collectors were Rob Mrowicki, Patrick Adkins, Joanna Harley and Rachel Brittain (Marine Biological Association). The specimen was identified by Rachel Brittain, based on gross morphology and DNA barcoding. Samples taken from the animal were preserved in liquid nitrogen. The fish was first anaesthetised and then overdosed using Aquased (2-phenoxyethanol). Destruction of the brain was used as a secondary method to ensure the animal was deceased before tissue sampling took place as in accordance with Schedule 1 methodology under the home office licence. Samples taken from the animal were preserved on dry ice.

The workflow for high molecular weight (HMW) DNA extraction at the Wellcome Sanger Institute (WSI) includes a sequence of core procedures: sample preparation; sample homogenisation, DNA extraction, fragmentation, and clean-up. In sample preparation, the fMicVar1 sample was weighed and dissected on dry ice ( Jay *et al.*, 2023). For sample homogenisation, muscle tissue was cryogenically disrupted using the Covaris cryoPREP ^®^ Automated Dry Pulverizer ( Narváez-Gómez *et al.*, 2023). HMW DNA was extracted using the Automated MagAttract v1 protocol ( Sheerin *et al.*, 2023). DNA was sheared into an average fragment size of 12–20 kb in a Megaruptor 3 system with speed setting 30 ( Todorovic *et al.*, 2023). Sheared DNA was purified by solid-phase reversible immobilisation ( Strickland *et al.*, 2023): in brief, the method employs a 1.8X ratio of AMPure PB beads to sample to eliminate shorter fragments and concentrate the DNA. The concentration of the sheared and purified DNA was assessed using a Nanodrop spectrophotometer and Qubit Fluorometer and Qubit dsDNA High Sensitivity Assay kit. Fragment size distribution was evaluated by running the sample on the FemtoPulse system.

RNA was extracted from muscle tissue of fMicVar1 in the Tree of Life Laboratory at the WSI using the RNA Extraction: Automated MagMax™ *mir*Vana protocol ( do Amaral *et al.*, 2023). The RNA concentration was assessed using a Nanodrop spectrophotometer and a Qubit Fluorometer using the Qubit RNA Broad-Range Assay kit. Analysis of the integrity of the RNA was done using the Agilent RNA 6000 Pico Kit and Eukaryotic Total RNA assay.

Protocols developed by the WSI Tree of Life laboratory are publicly available on protocols.io ( Denton *et al.*, 2023).

### Sequencing

Pacific Biosciences HiFi circular consensus DNA sequencing libraries were constructed according to the manufacturers’ instructions. Poly(A) RNA-Seq libraries were constructed using the NEB Ultra II RNA Library Prep kit. DNA and RNA sequencing was performed by the Scientific Operations core at the WSI on Pacific Biosciences SEQUEL II (HiFi) and Illumina NovaSeq 6000 (RNA-Seq) instruments. Hi-C data were also generated from muscle and gill tissue of fMicVar1 using the Arima2 kit and sequenced on the Illumina NovaSeq 6000, Illumina NovaSeq 6000 instrument.

### Genome assembly, curation and evaluation

Assembly was carried out with Hifiasm ( Cheng *et al.*, 2021) and haplotypic duplication was identified and removed with purge_dups ( Guan *et al.*, 2020). The assembly was then scaffolded with Hi-C data ( Rao *et al.*, 2014) using YaHS ( Zhou *et al.*, 2023). The assembly was checked for contamination and corrected using the gEVAL system ( Chow *et al.*, 2016) as described previously ( Howe *et al.*, 2021). Manual curation was performed using gEVAL, HiGlass ( Kerpedjiev *et al.*, 2018) and PretextView ( Harry, 2022). The mitochondrial genome was assembled using MitoHiFi ( Uliano-Silva *et al.*, 2023), which runs MitoFinder ( Allio *et al.*, 2020) or MITOS ( Bernt *et al.*, 2013) and uses these annotations to select the final mitochondrial contig and to ensure the general quality of the sequence.

A Hi-C map for the final assembly was produced using bwa-mem2 ( Vasimuddin *et al.*, 2019) in the Cooler file format ( Abdennur & Mirny, 2020). To assess the assembly metrics, the *k*-mer completeness and QV consensus quality values were calculated in Merqury ( Rhie *et al.*, 2020). This work was done using Nextflow ( Di Tommaso *et al.*, 2017) DSL2 pipelines “sanger-tol/readmapping” ( Surana *et al.*, 2023a) and “sanger-tol/genomenote” ( Surana *et al.*, 2023b). The genome was analysed within the BlobToolKit environment ( Challis *et al.*, 2020) and BUSCO scores ( Manni *et al.*, 2021; Simão *et al.*, 2015) were calculated.

Table 3 contains a list of relevant software tool versions and sources.

**Table 3.** Software tools: versions and sources.

Software tool	Version	Source
BlobToolKit	4.2.1	https://github.com/blobtoolkit/ blobtoolkit
BUSCO	5.3.2	https://gitlab.com/ezlab/busco
Hifiasm	0.16.1-r375	https://github.com/chhylp123/ hifiasm
HiGlass	1.11.6	https://github.com/higlass/ higlass
Merqury	MerquryFK	https://github.com/ thegenemyers/MERQURY.FK
MitoHiFi	3	https://github.com/ marcelauliano/MitoHiFi
PretextView	0.2	https://github.com/wtsi-hpag/ PretextView
purge_dups	1.2.5	https://github.com/dfguan/ purge_dups
sanger-tol/ genomenote	v1.0	https://github.com/sanger-tol/ genomenote
sanger-tol/ readmapping	1.1.0	https://github.com/sanger-tol/ readmapping/tree/1.1.0
YaHS	1.2a.2	https://github.com/c-zhou/yahs

### Wellcome Sanger Institute – Legal and Governance

The materials that have contributed to this genome note have been supplied by a Darwin Tree of Life Partner. The submission of materials by a Darwin Tree of Life Partner is subject to the **‘Darwin Tree of Life Project Sampling Code of Practice’**, which can be found in full on the Darwin Tree of Life website here. By agreeing with and signing up to the Sampling Code of Practice, the Darwin Tree of Life Partner agrees they will meet the legal and ethical requirements and standards set out within this document in respect of all samples acquired for, and supplied to, the Darwin Tree of Life Project.

Further, the Wellcome Sanger Institute employs a process whereby due diligence is carried out proportionate to the nature of the materials themselves, and the circumstances under which they have been/are to be collected and provided for use. The purpose of this is to address and mitigate any potential legal and/or ethical implications of receipt and use of the materials as part of the research project, and to ensure that in doing so we align with best practice wherever possible. The overarching areas of consideration are:

•     Ethical review of provenance and sourcing of the material

•     Legality of collection, transfer and use (national and international)

Each transfer of samples is further undertaken according to a Research Collaboration Agreement or Material Transfer Agreement entered into by the Darwin Tree of Life Partner, Genome Research Limited (operating as the Wellcome Sanger Institute), and in some circumstances other Darwin Tree of Life collaborators.

## Data Availability

European Nucleotide Archive: *Microchirus variegatus*. Accession number PRJEB64068; https://identifiers.org/ena.embl/PRJEB64068 ( Wellcome Sanger Institute, 2023). The genome sequence is released openly for reuse. The *Microchirus variegatus* genome sequencing initiative is part of the Darwin Tree of Life (DToL) project. All raw sequence data and the assembly have been deposited in INSDC databases. The genome will be annotated using available RNA-Seq data and presented through the Ensembl pipeline at the European Bioinformatics Institute. Raw data and assembly accession identifiers are reported in Table 1.

## Author information

Members of the Marine Biological Association Genome Acquisition Lab are listed here: https://doi.org/10.5281/zenodo.8382513.

Members of the Darwin Tree of Life Barcoding collective are listed here: https://doi.org/10.5281/zenodo.4893703.

Members of the Wellcome Sanger Institute Tree of Life Management, Samples and Laboratory team are listed here: https://doi.org/10.5281/zenodo.10066175.

Members of Wellcome Sanger Institute Scientific Operations: Sequencing Operations are listed here: https://doi.org/10.5281/zenodo.10043364.

Members of the Wellcome Sanger Institute Tree of Life Core Informatics team are listed here: https://doi.org/10.5281/zenodo.10066637.

Members of the Tree of Life Core Informatics collective are listed here: https://doi.org/10.5281/zenodo.5013541.

Members of the Darwin Tree of Life Consortium are listed here: https://doi.org/10.5281/zenodo.4783558.
