# Supplementary material for: The genome sequence of the thickback sole, Microchirus variegatus (Donovan, 1808)
Source: Wellcome Open Res. 2024 Mar 19;9:152. [Version 1] doi: 10.12688/wellcomeopenres.21139.1 (PMC11208857; doi:10.12688/wellcomeopenres.21139.1)
Supplement: Peer review history

# Reviewer report: Reviewer response for version 1

**DOI:** 10.21956/wellcomeopenres.23383.r84566

**Published:** 2024-06-26

**Authors:** Jianguo Lu

**Affiliations:** 1 Zhuhai Campus, Sun Yat-Sen University, Tangjiawan, China

The manuscript titled "The genome sequence of the thickback sole, *Microchirus variegatus *(Donovan, 1808)" presents a comprehensive genomic assembly from an individual female specimen of this lesser-known flatfish species. Spanning 724.7 megabases, the assembly is meticulously organized into 23 chromosomal pseudomolecules, reflecting a commendable effort in achieving high-resolution genomic structure elucidation. Additionally, the inclusion of the 17.42-kilobase mitochondrial genome underscores the completeness of the presented dataset. The methodology is suitable and overall, very well described. I have noticed only few small inconsistencies:

1. Table 1 of the manuscript lists the Genome Assembly with a 'Number of contigs' as 642 for the accession GCA_963457635.1. However, the same accession on NCBI database cites a count of 641 contigs. Please reconcile this discrepancy and confirm the accurate number of contigs in the assembly.
2. In the data note, while the full name 'Marine Biological Association' is correctly presented in the author affiliation details, its initial appearance in the main body of the text omits the expansion from the abbreviation 'MBA'. This omission has the potential to cause confusion for readers unfamiliar with the acronym. Hence, it is recommended to introduce the full designation 'Marine Biological Association (MBA)' at its first use in the text to promote clarity and accessibility.
3. The same data in the manuscript does not use the same decimal intensive reading. Table 1 cites the 'Span (Mb)' as 724.7, whereas Figure 2 mentions 725 megabases. Harmonizing these figures to a consistent level of precision throughout would contribute to a smoother reading experience and ensure absolute clarity. While this adjustment is not a strict necessity, implementing it could refine the manuscript's overall coherence.
4. The 'Genome assembly, curation, and evaluation' phase encompassed a suite of software tools. While Table 3 provides version details and source links for several of these tools, there appears to be an omission regarding others instrumental in the process.

Are sufficient details of methods and materials provided to allow replication by others?

Yes

Is the rationale for creating the dataset(s) clearly described?

Yes

Are the datasets clearly presented in a useable and accessible format?

Yes

Are the protocols appropriate and is the work technically sound?

Yes

Reviewer Expertise:

NA

I confirm that I have read this submission and believe that I have an appropriate level of expertise to confirm that it is of an acceptable scientific standard.

# Reviewer report: Reviewer response for version 1

**DOI:** 10.21956/wellcomeopenres.23383.r82288

**Published:** 2024-06-07

**Authors:** Sandhya Sukumaran

**Affiliations:** 1 ICAR-Central Marine Fisheries Research Institute, Kochi, India

Title of the paper : The genome sequence of the thickback sole, *Microchirus*

*variegatus *(Donovan, 1808)

 The article describes the whole genome sequencing of the thickback sole, Microchirus variegatus. It is an excellent piece of research that is carefully carried out. The reported genome assembly at the chromosome level is of high quality as shown by the BUSCO values. The sequencing coverage is also good. I just need some clarification.

 1. Is there any information regarding genome size of *Microchirus variegatus* estimated using flow cytometry? If yes, then what percentage of genome has been covered in this assembly?

 2. It will be good if you can give details regarding the repeat content of the genome and the major repeat units represented in the genome.

 I would recommend accepting this paper after addressing these comments.

Are sufficient details of methods and materials provided to allow replication by others?

Yes

Is the rationale for creating the dataset(s) clearly described?

Yes

Are the datasets clearly presented in a useable and accessible format?

Yes

Are the protocols appropriate and is the work technically sound?

Yes

Reviewer Expertise:

Fish genetics and genomics

I confirm that I have read this submission and believe that I have an appropriate level of expertise to confirm that it is of an acceptable scientific standard.
